# A novel PMP22 insertion mutation causing Charcot–Marie–Tooth disease type 3: A case report

**DOI:** 10.1097/MD.0000000000025163

**Published:** 2021-03-19

**Authors:** Liang Han, Yanjing Huang, Yuan Nie, Jing Li, Gang Chen, Shenghao Tu, Pan Shen, Chao Chen

**Affiliations:** a Department of Integrated Traditional Chinese and Western Medicine, Tongji hospital, Tongji Medical College, Huazhong University of Science and Technology, 1095 Jiefang Avenue, Wuhan; b Rehabilitation Center, Qijiang District Hospital of Traditional Chinese Medicine, 50 Dashi Road of Wenlong Avenue, Chongqing; c Department of Orthopaedics, Union Hospital, Tongji Medical College, Huazhong University of Science and Technology, 1277 Jiefang Avenue, Wuhan, China.

**Keywords:** Charcot–Marie–Tooth disease (CMT), Dejerine–Sottas syndrome (DSS), demyelinating diseases, insertion mutation, PMP22, whole-exome sequencing (WES)

## Abstract

**Rationale:**

Charcot–Marie–Tooth disease (CMT) is a group of hereditary neuropathies with clinical features of muscle atrophy, sensory loss, and foot deformities. CMT is related to a number of genes, such as peripheral myelin protein 22 gene (*PMP22*). Missense mutations, small deletion mutations, and duplications of *PMP22* are common in CMT patients, but few insertion mutation cases of *PMP22* have been reported.

**Patient concerns:**

A 26-year-old male patient with the complaint of general weakness, peroneal atrophy, and deformities in the extremities visited our hospital. The patient was born with bilateral thumbs and feet dystonia. Additionally, delayed feet arch development and delayed walking was observed when he was a child.

**Diagnosis:**

Using whole-exome sequencing and electrophysiological test, we identified a novel insertion mutation of *PMP22* (NM_153322, c.54_55insGTGCTG, p.(L19delinsVLL)) in a 26-year-old male patient with peroneal atrophy and nerve conduction was not elicited in electromyography (EMG) study. The Protein Variation Effect Analyzer (*PROVEAN*) program analysis predicted that the variant is likely to be “deleterious.” *SWISS-MODEL* program predicted that alpha helix in original location was disrupted by inserted 6 bases, which may account for the occurrence of CMT3.

**Interventions:**

The patient received symptomatic and supportive treatments, and routine rehabilitation exercises during hospitalization.

**Outcomes:**

The condition of the patient was improved, but the disease could not be cured. At 1- and 3-months follow-up, manifestations of the patient were unchanged, and he could take care of himself.

**Lessons:**

Our findings link a novel *PMP22* mutation with a clinical diagnosis of CMT3. The link between gene variation and CMT phenotype may help to reveal the structure and function of PMP22 protein and the pathogenesis of CMT. This study adds further support to the heterogeneity of PMP22 related CMT and provides solid functional evidence for the pathogenicity of the p.(L19delinsVLL) *PMP22* variant. Moreover, with the development of high-throughput sequencing technology, the combination of next-generation sequencing (NGS) and conventional Sanger sequencing is becoming one of the comprehensive, inexpensive, and convenient tools for genetic diagnosis of CMT.

## 1 Introduction

Charcot–Marie–Tooth disease (CMT), also known as hereditary motor and sensory peripheral neuropathy, predominantly involves distal muscle weakness and atrophy, and sensory loss. The 2 main subtypes of CMT are the demyelinating form (CMT1, CMT3, and CMT4) and the axonal form (CMT2), and these are distinguished based on neurophysiological differences. These symptoms are related to genetic changes.^[1]^ CMT1A is the most common subtype of CMT1 and is caused by peripheral myelin protein 22 gene (*PMP22*) duplication. In addition, CMT1 is also linked to point mutations in *PMP22*, and other mutations in myelin protein zero (*MPZ*) and early growth response 2 (*EGR2*) and other genes. In contrast, CMT3, also known as Dejerine–Sottas syndrome, is rarer and more severe than CMT1.^[2]^ CMT3 is distinctively characterized by early onset, by increased cerebrospinal fluid (CSF) protein levels, and by very slow nerve conduction velocities.

*PMP22* encodes the PMP22 protein, which is mainly expressed in Schwann cells and plays a crucial role in the formation and maintenance of compact myelin. Patients with CMT1A are usually associated with *PMP22* overexpression, which is related to *PMP22* duplication. Overexpression of *PMP22* in Schwann cells induces inherited demyelination in patients with abnormal *PMP22.*^[3]^*PMP22* duplications account for approximately 50% of all CMT cases. In contrast, *PMP22* small mutations, including missense mutations, small deletion mutations and small deletion mutations, related CMT3 and CMT1E are rarer than *PMP22* duplications related CMT1A.^[4]^

Here, we report a 26-year-old male Chinese patient with general weakness, peroneal atrophy, nerve conduction was not elicited in electromyography (EMG) study, deformities in the extremities, and other CMT3 phenotypes, with a novel heterozygous mutation (NM_153322, c.54_55insGTGCTG, p.(L19delinsVLL)) of *PMP22*. This previously unreported mutation might be associated with early onset and severe demyelination in CMT.

## 2 Methods

### 2.1 Clinical investigations

Analyses of routine blood and urine, and stool, hepatic and renal function, CSF, and serum creatine kinase, and tumor markers (alpha-fetoprotein, carcinoembryonic antigen, carbohydrate antigen 19-9, prostate-specific antigen, cytokeratin-19 fragments, squamous cell carcinoma antigen, and thyroid-stimulating hormone) were performed. Analyses of EMG and magnetic resonance (MR) imaging of the cranium and extremities were also performed.

Analysis of autoimmune diseases antibodies (anti-nuclear antibodies, anti-double stranded DNA antibodies, anti-chromatin antibodies, anti-ribonucleoprotein antibodies, anti-Smith antibodies, anti-Sjögren's-syndrome type A autoantibodies, anti-Sjögren's-syndrome type B autoantibodies, anti-ro52 antibodies, anti-Scl-70 antibodies, anti-Jo-1 antibodies, anti-CENP-B antibodies, anti-CENP-P antibodies, rheumatoid factor, anti-cyclic citrullinated peptide antibodies, anti-keratin antibodies, anti-glomerular basement membrane antibodies, anti-myeloperoxidase antineutrophil cytoplasmic antibodies, anti-proteinase 3 antineutrophil cytoplasmic antibodies, perinuclear antineutrophil cytoplasmic antibodies, cytoplasmic antineutrophil cytoplasmic antibodies) was also performed.

### 2.2 Genetic analysis

DNA was extracted from a peripheral blood sample for high-throughput whole-exome sequencing which was performed at the Genetic Diagnosis Center of Tongji Hospital. Common single nucleotide polymorphisms in the UCSC database, common mutations in The Single Nucleotide Polymorphism Database (dbSNP) and International Genome Sample Resource (IGSR), and other synonymous mutations or intron mutations were filtered out. Finally, the positive mutation was confirmed by Sanger sequencing. To determine if pathogenicity of the variant could be readily predicted, we used the Protein Variation Effect Analyzer (*PROVEAN*) (http://provean.jcvi.org/) to evaluate variants to determine pathogenicity. 3D structures of the PMP22 wild-type and the mutant in this study were created based on mouse claudin-15 (PDB code 4P79) via *SWISS-MODEL* program (https://swissmodel.expasy.org/).^[5]^

## 3 Case report

### 3.1 Clinical findings

A 26-year-old man with general weakness, peroneal atrophy, and deformities in the extremities presented at our hospital. The patient's parents and 2 elder sisters were healthy and free of any neurological diseases. The patient reported that he was born at term in his home by normal spontaneous vaginal delivery, and the birth was assisted by a midwife. His birth weight, height, and head circumference were not noted. The patient's bilateral thumbs and feet were weak at birth. The patient could walk by the age of 6 years, although he had an unstable gait and fallen arches in his feet. The arches in his feet developed gradually. The patient underwent bilateral straightening of the feet at a local hospital at the age of 16, which resulted in greater improvement in his right foot. The patients had visited multiple hospitals, but the etiology remained unknown. His parents reported that he had no history of seizures. The patient had a history of chronic gastritis for 10 years, while he was at junior high school; however, he had no other previous history of any disease and was not addicted to either alcohol or tobacco.

We examined the patient in August 2019. His vital signs, measured at the time of admission, were normal (body temperature: 36.5°C; pulse rate: 104 beats/minute; respiratory frequency: 20 breaths/minute; blood pressure: 123/85 mm Hg). The patient walked with a myopathic gait because of weakness in the lower extremities. We observed variable scoliosis, deformities of both thumbs and both feet (pes cavus), and symmetric atrophy of the bilateral thenar muscles, bilateral hypothenar muscles, and bilateral muscles of under knees. Muscle strength in the bilateral upper distal limbs, bilateral upper proximal limbs, bilateral lower distal limbs, and bilateral lower proximal limbs was grade IV, grade IV+, grade IV, and grade IV−, respectively. Tendon areflexia in the extremities, deep sensory dysfunction of the right leg, and the Romberg sign were all also observed.

### 3.2 Laboratory findings

Results from the CSF protein test were positive, and further laboratory tests showed that albumin, IgA, and IgG were elevated in the CSF. The concentration of serum neuron-specific enolase was slightly elevated (24.29 μg/L; ≤16.30 μg/L is normal), and serum creatine kinase was very elevated (529 U/L; ≤190 U/L is normal). Analyses of routine blood and urine, stool, as well as hepatic and renal function, and tumor markers showed no clinically significant abnormal values related to the syndrome. Additionally, all antibodies of autoimmune diseases were negative.

### 3.3 Magnetic resonance and electrophysiological findings

**Figure 1:**
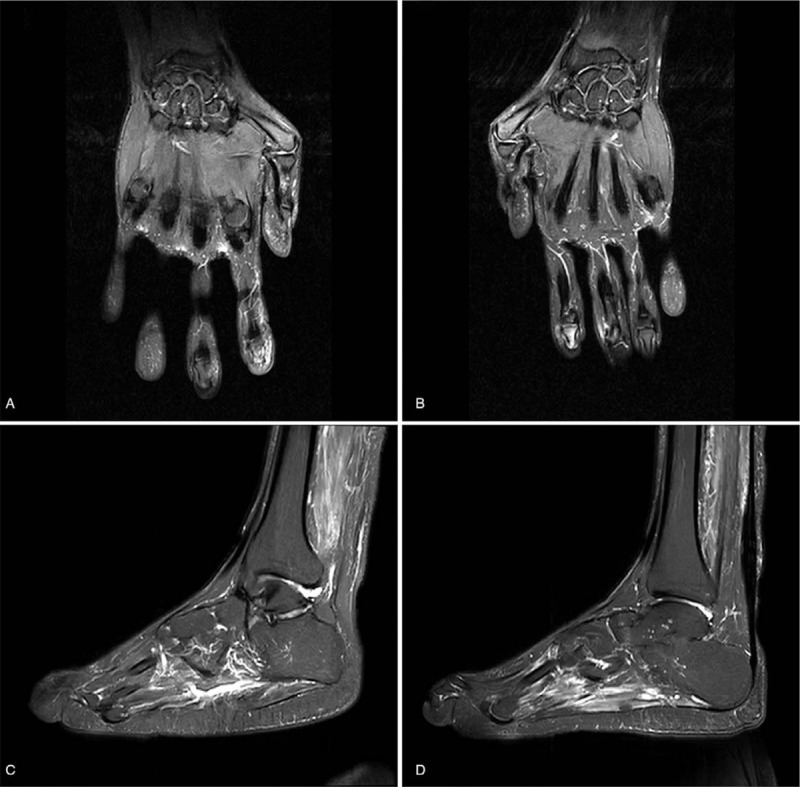
Magnetic resonance (MR) imaging T2-weighted images (T2WI) of bilateral hands and feet (a–d) revealed joint effusions, muscles and soft tissues injury in bilateral hands and feet. (a) MR imaging of left hand. (b) MR imaging of right hand. (c) MR imaging of left foot. (d) MR imaging of right foot.

Cranial routine MR showed no obvious abnormalities in the brain. Routine MR of both the right and left hand demonstrated marrow edema at the end of the first metacarpal bone, malunions of the first metacarpophalangeal joint, and a small number of effusions in the first metacarpophalangeal and wrist joints. Routine MR of the left foot revealed joint effusions in the ankle, intertarsal, tarsometatarsal, and metatarsophalangeal joints, as well as a slight narrowing of both the talocalcaneal and calcaneocuboid joint spaces. In addition, edema of the posterior tibial muscle, sole muscles, and fascia were observed, as well as swelling of the ankle joint and soft tissues around the foot. Routine MR of the right foot revealed joint effusions in the ankle, taloscaphoid, talocalcaneonavicular, and metatarsophalangeal joints, as well as swelling of the ankle joint and soft tissues around the foot, and severe narrowing of the talocalcaneal joint space. MR images of bilateral hands and feet are shown in Figure 1.

EMG results revealed that nerve conduction was not elicited in the motor and sensory bilateral median nerves, bilateral ulnar nerves, bilateral tibial nerves, and common peroneal nerves. Spontaneous potential was observed from the right anterior tibial muscle, and EMG results revealed neurogenic damage. Spontaneous potentials were also observed from the right abductor pollicis brevis, but motor unit potentials were not recorded. Detailed EMG results are shown in Tables 1 and 2. Above EMG results indicated that the patient has a polyneuropathy.

**Table 1:** Motor and sensory nerves conduction in the patient's extremities.

	Latency (ms)	Amplitude (mv)	Distance (mm)	Conduction velocity (m/s)
Motor ulnar left				
Wrist-ADM	Not elicited	NA	NA	NA
Elbow-wrist	Not elicited	NA	NA	NA
Motor ulnar right				
Wrist-ADM	Not elicited	NA	NA	NA
Elbow-wrist	Not elicited	NA	NA	NA
Motor median left				
Wrist-APB	Not elicited	NA	NA	NA
Motor median right				
Wrist-APB	Not elicited	NA	NA	NA
Elbow-wrist	Not elicited	NA	NA	NA
Motor tibial left				
Ankle-AH	Not elicited	NA	NA	NA
Knee-ankle	Not elicited	NA	NA	NA
Motor tibial right				
Ankle-AH	Not elicited	NA	NA	NA
Knee-ankle	Not elicited	NA	NA	NA
Motor peroneal left				
Ankle-EDB	Not elicited	NA	NA	NA
Fibular head-ankle	Not elicited	NA	NA	NA
Motor peroneal right				
Ankle-EDB	Not elicited	NA	NA	NA
Fibular head-ankle	Not elicited	NA	NA	NA
Sensory ulnar left				
Digit V-wrist	Not elicited	NA	NA	NA
Sensory ulnar right				
Digit V-wrist	Not elicited	NA	NA	NA
Sensory median left				
Digit II-wrist	Not elicited	NA	NA	NA
Sensory median right				
Digit II-wrist	Not elicited	NA	NA	NA
Sensory superficial peroneal left				
Calf-Med. Dor. Cutan.	Not elicited	NA	NA	NA
Sensory superficial peroneal right				
Calf-Med. Dor. Cutan.	Not elicited	NA	NA	NA
Sensory sural left				
Mid calf-ankle	Not elicited	NA	NA	NA
Sensory sural right				
Mid calf-ankle	Not elicited	NA	NA	NA

ADM = abductor digiti minimi, AH = abductor hallucis, APB = abductor pollicis brevis, EDB = extensor digitorum brevis, m/s = meter per second, Med. Dor. Cutan. = medial dorsal cutaneous, mm = millimeter, ms = millisecond, mv = millivolt, NA = not available.

**Table 2:** Electromyography findings in the patient's right tibialis anterior and right abductor.

	Spontaneous activity		Voluntary activity					
	Fib	PSW	Amp	Dur	Poly	Stabil	IP	Annotation
Right tibialis anterior	2/10	3/10	+	++	+++	Normal	Simple phase	NA
Right abductor pollicis brevis	0/10	2/10	NA	NA	NA	NA	NA	NA

Amp = amplitude, Dur = duration, Fib = fibrillation, IP = interference pattern, NA = not available, Poly = polyphase, PSW = positive sharp waves, Stabil = stability.

### 3.4 Genetic findings

**Figure 2:**
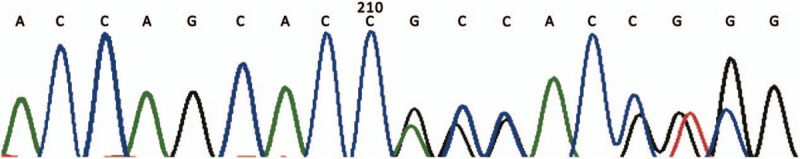
Sanger sequencing chromatogram confirmed the results from whole-exome sequencing. The chromatogram proved that the patient has a heterozygous insertion mutation in PMP22 (NM_153322, c.54_55insGTGCTG).

**Figure 3:**
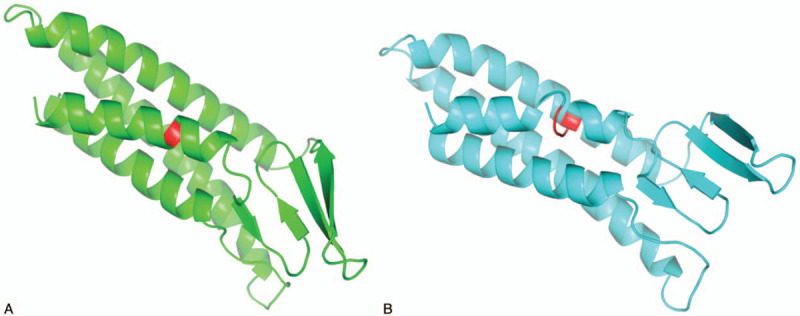
Compared with wild-type protein, c.54_55insGTGCTG made the leucine at position 19 has been substituted with 3 amino acid residues (Val-Leu-Leu). Wild-type and mutation structure of PMP22 predicted by SWISS-MODEL program indicated the alpha helix disruption caused by the change of amino acid sequence. (a) Wild-type PMP22 predicted 3D structure. (b) Mutation PMP22 predicted 3D structure. Red regions represent deletion amino acid residue in (a) or insertion amino acid residues in (b), respectively.

Genetic analysis of the patient's blood sample revealed a heterozygous mutation in *PMP22* (NM_153322, c.54_55insGTGCTG, p.(L19delinsVLL)), which has uncertain significance according to the American College of Medical Genetics and Genomics (ACMG).^[6]^ Other than that, no other pathogenic mutations were found in WES. The verification results of the positive mutation using Sanger sequencing are shown in Figure 2. The *PROVEAN* program analyzed the variant to be “deleterious”, with a score of -9.286.^[7]^ The wild-type and mutation structure of PMP22 were created via *SWISS-MODEL* program (Fig. 3a and b). By comparing Figure 3a and Figure 3b, it is easy to find that one of alpha helixes structure was disrupted according to the predicted 3D structures.

## 4 Discussion

CMT is one of the most prevalent inherited peripheral neuropathies that affect motor and sensory neurons. CMT was first described in 1886 by Charcot, Marie, and Tooth together.^[8]^ CMT can be divided into 2 main categories based on patients’ nerve conduction velocities and nerve pathology. One is the demyelinating form, which has slower nerve conduction velocities and distinct myelin abnormalities, such as onion bulb formations. The other kind is the axonal form of CMT, which involves relatively preserved or slightly slowed nerve conduction velocities, and chronic axonal degeneration and regeneration at nerve biopsy. An upper-limb motor nerve conduction velocity < 38 m/second is considered to indicate the demyelinating form of CMT.^[4]^ However, increasing reports of relationship between the clinical phenotypes and genetic mutations blurs the boundaries of CMT subtypes. Considering the lack of uniform classification criteria of CMT, CMT3, the old name of the disease was still used in our study.

CMT3, also known as DSS, was firstly described by Dejerine and Sottas at 1893. Its phenotypes are genetically heterogeneous, which is the same as other CMT subtypes. However, CMT3 has a series of distinctive phenotypes. Specifically, onset of CMT3 usually occurs between infancy and early childhood, which is generally earlier than in patients with CMT1. Patients with CMT3 also obtain worse results in electromyography and have severely reduced nerve conduction velocities (below 12 m/second). Onion bulb formation, segmental demyelination, and remyelination are also observed by nerve biopsy in CMT3 patients.^[4]^

The relation between phenotypes of CMT and the mutated genes is complicated, and there are not one-to-one correspondences. As the most common type of CMT, CMT1 is linked with *PMP22*,^[9]^*MPZ*,^[10]^*EGR2*,^[11]^ lipopolysaccharide-induced tumor necrosis factor-alpha factor (*LITAF*),^[12]^ and neurofilament light chain polypeptide genes (*NEFL*),^[13]^ while CMT1A is mainly caused by a 1.5 Mb duplication on chromosome 17p11.2 that includes the *PMP22* gene.^[14]^ However, some patients with mutations of *PMP2*, *MPZ*, periaxin gene (*PRX*), or *EGR2* may show more severe CMT subtype, that is, CMT3.^[10,15–17]^

*PMP22* is a 40 kb gene consisting of 6 exons that are conserved in humans and rodents. This gene is located within chromosome 17p11.2 in the human genome. The predicted protein structure of PMP22 has 4 transmembrane domains, 2 extracellular domains, and 1 intracellular domain.^[18]^ Aberrant *PMP22* is one of the most important pathogenic genes in CMT. Approximately 50% of all CMT cases are caused by mutations in *PMP22*, and abnormalities of *PMP22*-related neuropathies can be principally divided into 2 types. *PMP22* duplications or point mutations are linked with CMT1A, while other small mutation types (missense mutations, small deletion mutations, and small deletion mutations) of *PMP22* are linked with CMT1E and CMT3. Moreover, *PMP22* small mutations are also related to hereditary neuropathy with pressure palsies (HNPP), an inherited peripheral neuropathy caused by a deletion of *PMP22* or pathogenic variant of *PMP22*. *PMP22* small insertion mutations are rare and only 6 cases have been reported according to the Human Gene Mutation Database (HGMD) (http://www.hgmd.cf.ac.uk). Among them, only 2 cases are related to CMT phenotype,^[19,20]^ and other insertion mutations are related to HNPP phenotype.^[21–23]^

The pathogenesis of *PMP22* mutation related CMT remains unclear. Presumably, PMP22 duplications eventually lead to overexpression of PMP22 protein, which causes the CMT1A phenotype.^[24]^ However, the pathogenesis of *PMP22* duplications related CMT1A seems not same as that in CMT patients with *PMP22* small mutations.^[25]^ Moreover, it was also recognized that PMP22 is important to both the sealing of myelinated nerve fibers and their protection from mechanical stresses, and *PMP22* point mutation in Trembler-J mice exhibits impaired mechanical integrity.^[26]^

Here, we report a new insertion mutation of *PMP22* (NM_153322, c.54_55insGTGCTG, p.(L19delinsVLL)) that was discovered using next-generation sequencing and was confirmed using Sanger sequencing. This mutation affects the first transmembrane domain of PMP22. The *PROVEAN* program is a tool to predict the functional effect of amino acid substitutions and indels.^[7]^ It is useful for filtering sequence variants to identify nonsynonymous or indel variants that are predicted to be functionally important. Variants with a score equal to or below −2.5 are considered “deleterious,” otherwise, it was considered “neutral.” Computational analysis predicted that the variant is likely to have pathogenic significance. Moreover, structural prediction of the PMP22 suggested that one of alpha helixes in transmembrane region was disrupted by insertion mutation. This aberrant secondary structure of PMP22 might be a possible cause of the CMT3 phenotype.

In this case, series of muscle abnormalities both at birth and in childhood indicated that there might be genetic changes, and WES result confirmed this. The abnormal *PMP22* variant might be linked with a phenotype of distal muscle weakness and atrophy, scoliosis, deformities of both thumbs and both feet, and elevated CSF protein and serum creatine kinase. Nerve conduction could not be elicited in EMG testing, indicating serious demyelination and axonal degeneration. In addition, serum creatine kinase and serum neuron-specific enolase were elevated, and MR results revealed severe muscle damage in the hands and legs. Negative antibodies of autoimmune diseases showed it might not be a kind of autoimmune diseases. In this case, it is not hard to diagnose suspected CMT just based on the history, symptoms, and electrophysiological test results. For neuromuscular genetic diseases, diagnosis by clinical features alone is highly unreliable, thus, pathological examination and genetic test were recommend to diagnosis.^[4]^ However, the patient and his parents were not willing to allow nerve and muscle biopsies, so we could not reveal the pathological alterations. Fortunately, WES tests results provide solid evidence for the final diagnosis. Although there was no direct evidence, the heterozygous mutation, significant phenotype, and healthy parents all suggest that this patient had a *de novo* autosomal dominant mutation.

Genetic testing is the gold standard to confirm the diagnosis of hereditary diseases. Previously, sequencing of several suspected genes selected by physician experiences or certain procedures according to patients’ clinical features is necessary to diagnosis CMT.^[27]^ However, the evolvement of high-throughput sequencing technology is changing this. In our case, WES and Sanger sequencing offered credible molecular evidence for a patient with CMT3 phenotype rapidly and inexpensively, which could be helpful to future individual targeted treatment.^[28]^ Meanwhile, the lower and lower price of high-throughput technology make it more easily accepted by patients. Taken together, WES or whole-genome sequencing (WGS) in combination with Sanger sequencing would be more suitable for CMT and other similar disease whose relationship between clinical phenotype and genetic changes is complex and confused, and this technique is worthy of clinical promotion.

There is no effective drug treatment for CMT at present, and functional exercises are still important for CMT patients.^[29]^ Since we found that there were peripheral nerve injuries, symptomatic treatment agents which could improve the symptoms of neuropathy, including mecobalamin injection, cattle encephalon glycoside and ignotin injection, and 2 Chinese patent drugs (placenta polypeptide injection and bozhi glycopeptide injection), were given.^[30–32]^ When we were aware that patient presented a clinical CMT3 phenotype, functional exercising was considered as a primary treatment. Gratifyingly, the patient kept up with the routine rehabilitation exercising and it improved his weakness slightly. The patient could take care of himself during the hospitalization period, and he was discharged from our hospital after 14 days medical screenings and treatment. We advise the patient to do functional training in home and Department of Rehabilitation of local hospitals institutions regularly. The patient told us that his manifestations were unchanged, and he could care himself independently at 1- and 3-months follow-up, respectively.

## 5 Conclusions

CMT is one of the most common peripheral neuropathies caused by genetic changes. Complex clinical phenotype and high genetic heterogeneity hamper the comprehensive understandings of its pathogenesis. In this study, we present a patient with CMT3 associated with a new insertion mutation of *PMP22*, which enlarged the genetic spectrum of CMT correlated with PMP22 protein. *PMP22* mutations were usually considered to be either duplications or small mutations, 4 or more bases insertion mutation has never been reported. Considering that the pathogenesis of CMT caused by abnormal PMP22 protein is still unclear, more cases and basic experiments are needed to understand how this insertion mutation may cause a severe CMT phenotype. The combination of high-throughput sequencing and Sanger sequencing was crucial for final diagnosis, which deserves to be considered for the diagnosis of CMT and other similar inherited diseases.

## Acknowledgments

We would like to express our sincere gratitude to the patient's understanding and participation in this study.

## Author contributions

**Conceptualization:** Chao Chen.

**Data curation:** Liang Han, Yanjing Huang, Yuan Nie.

**Formal analysis:** Liang Han.

**Funding acquisition:** Jing Li, Gang Chen, Chao Chen.

**Investigation:** Liang Han, Yanjing Huang, Jing Li, Gang Chen.

**Methodology:** Liang Han, Chao Chen.

**Project administration:** Chao Chen.

**Resources:** Yanjing Huang, Yuan Nie, Pan Shen.

**Software:** Liang Han.

**Supervision:** Chao Chen.

**Visualization:** Liang Han.

**Writing – original draft:** Liang Han.

**Writing – review & editing:** Liang Han, Yanjing Huang, Jing Li, Gang Chen, Shenghao Tu, Pan Shen.
